# Flavonoid Naringenin Attenuates Oxidative Stress, Apoptosis and Improves Neurotrophic Effects in the Diabetic Rat Retina

**DOI:** 10.3390/nu9101161

**Published:** 2017-10-24

**Authors:** Dalia I. Al-Dosari, Mohammed M. Ahmed, Salim S. Al-Rejaie, Abdullah S. Alhomida, Mohammad S. Ola

**Affiliations:** 1 Department of Biochemistry, College of Science, King Saud University, Riyadh 11451, Saudi Arabia; Dalia.ib89@gmail.com (D.I.A.-D.); alhomida@ksu.edu.sa (A.S.A.); 2 Department of Pharmacology &Toxicology, College of Pharmacy, King Saud University, Riyadh 11451, Saudi Arabia; mmahmedd@ksu.edu.sa (M.M.A.); rejaie@ksu.edu.sa (S.S.A.-R.)

**Keywords:** diabetic retinopathy, flavonoid, naringenin, neurotrophic factor, oxidative stress, apoptosis

## Abstract

Diabetic retinopathy (DR) is one of the leading causes of decreased vision and blindness worldwide. Diabetes-induced oxidative stress is believed to be the key factor that initiates neuronal damage in the diabetic retina leading to DR. Experimental approaches to utilize dietary flavonoids, which possess both antidiabetic and antioxidant activities, might protect the retinal damage in diabetes. The aim of this study was to investigate the potential protective effects of naringenin in the retina of streptozotocin-induced diabetic rats. Diabetic rats were orally treated and untreated with naringenin (50 mg/kg/day) for five weeks and retinas were analyzed for markers of oxidative stress, apoptosis and neurotrophic factors. Systemic effects of naringenin treatments were also analyzed and compared with untreated groups. The results showed that elevated levels of thiobarbituric acid reactive substances (TBARs) and decreased level of glutathione (GSH) in diabetic rats were ameliorated with naringenin treatments. Moreover, decreased levels of neuroprotective factors (Brain derived neurotrophic factor (BDNF)), tropomyosin related kinase B (TrkB) and synaptophysin in diabetic retina were augmented with naringenin treatments. In addition, naringenin treatment ameliorated the levels of apoptosis regulatory proteins; B cell lymphoma 2 (Bcl-2), Bcl-2 associated X protein (Bax) and caspase-3 in the diabetic retina. Thus, the study demonstrates the beneficial effects of naringenin that possesses anti-diabetic, antioxidant and antiapoptotic properties, which may limit neurodegeneration by providing neurotrophic support to prevent retinal damage in diabetic retinopathy.

## 1. Introduction

Diabetic retinopathy (DR) is one of the most serious complications of diabetes mellitus and the leading cause of decreased vision and blindness in the developed countries. Numerous studies in experimental rodents and in diabetic subjects suggest that diabetes damages both neuronal and vascular components of the retina [1,2,3]. Furthermore, a large body of evidence suggest that neurons are damaged shortly after the onset of diabetes, which may trigger vascular injury that leads to diabetic retinopathy at the later stages of diabetes [3,4,5]. The exact pathophysiological mechanism of the neuro-vascular damage in the diabetic retina is still elusive. However, a number of research reports suggest that oxidative stress induced by diabetes is the central factor in initiating the cascade of retinal damage [6,7]. Diabetic induced altered metabolites exert an increase in the level of oxidative stress, which thereby may damage retinal cells [8,9]. These cell damaging effects are mainly caused by activation of apoptosis and inflammation, and also by lowering neurotrophic support due to increased oxidative stress, which thereby may initiate the development of lesions in diabetic retina [2,5,10].

Molecular mechanisms of retinal damage suggest that high oxidative stress causes profound damage to the diabetic retina by severe lipid peroxidation, protein oxidation, and oxidative DNA damage [8,11]. It has been reported that diabetic retina has lower levels of endogenous antioxidants glutathione, which makes retina vulnerable to damage [6,11]. In addition, high oxidative stress alters signal transduction and expression of pro/anti-apoptotic proteins [6,12]. Under diabetic conditions, oxidative stress induces apoptosis by damaging mitochondrial membrane that translocates proapoptotic protein Bcl-2 associated X protein (BAX) from cytosol to mitochondria and increases the release of cytochrome c which in turn activates caspases, while antiapoptotic Bcl-2 protein level are decreased [6]. Another key factor for neurovascular damage in the diabetic retina is the imbalance of neurotrophic factors [10,13,14]. Oxidative stress is considered the major factor that causes alternations in neurotrophic factors in the diabetic retina [15,16]. Brain derived neurotrophic factor (BDNF) plays an important role in neuronal survival and maintenance [2]. BDNF is initially synthesized in a precursor, proBDNF form, which later undergoes proteolytic cleavage intracellularly to produce the mature form. Several studies have reported decreased levels of BDNF and its downstream neuroprotective signaling through tropomyosin-related kinase B (TrkB) and synaptophysin in the retina of diabetic animals, which cause serious alterations in retinal function [11,15,17,18]. Moreover, increased ratio of proBDNF to BDNF promotes neuronal apoptosis and attenuates synaptic transmission [19]. Therefore, utilization of antioxidant defense strategies may counterbalance oxidative damage and thereby ameliorate neurotrophic imbalance and signaling to protect neurons in the diabetic retina.

A growing number of experimental evidence have emerged, which support the concept that flavonoids with their strong antioxidant activities ameliorate oxidative stress and thereby may prevent damage to the diabetic retina [11,20,21,22]. Flavonoids being antioxidant can ameliorate apoptosis, and neurotrophic factors, thereby may protect neuronal damage in the diabetic retina [11,21,23]. In this study, we utilized the flavonoid, naringenin to study the neuroprotective effects in the diabetic retina. Naringenin (2,3-dihydro-5,7-dihydroxy-2-(4-hydroxyphenyl)-4H-1-benzopyran-4-one) is a flavanone, flavonoid, abundantly present in citrus fruits. Naringenin has different pharmacological activities including antioxidant [24], antidiabetic [25], anti-inflammatory [26], and also possesses neuroprotective activities in different experimental models of rodents [27,28]. Recently, our group has reported neuro-protective effect of naringenin in the sciatic nerve of diabetic rats by its antioxidant and anti-inflammatory properties [29]. Despite of a number of beneficial effects of naringenin, to our knowledge, no studies have reported the neuroprotective effects in the diabetic retina. Therefore, in this study we investigated the potential protective effects of naringenin in the retina of streptozotocin-induced diabetic rats, by assessing various oxidative with apoptotic and neurodegenerative markers.

## 2. Methods

### 2.1. Animals and Experimental Model

Twelve weeks aged male Wistar albino rats, weighing 250–290 g were received from Experimental Animal Care Center (King Saud University, Riyadh, Saudi Arabia). Diabetes was induced by single intraperitoneal injection of streptozotocin (STZ) (Sigma, St. Louis, MO, USA) at a dose of 65 mg/kg body weight, dissolved in 0.1 mol/L citrate buffered solution (pH 4.5). Three days after STZ injection, blood glucose was measured and blood glucose >250 mg/dL was considered diabetic and included in the study. Naringenin treatment was started orally by gavage (50 mg/kg/day) after two weeks of STZ-injection and continued for five consecutive weeks. A total of 40 rats (*n* = 10) were randomly divided into four groups as follows; Group 1: Control rats treated with vehicle (C); Group 2: Control rats treated with naringenin (C + N); Group 3: Diabetic rats treated with vehicle (D); Group 4: Diabetic rats treated with naringenin (D + N). At the end of five weeks of treatments, animals were fasted overnight, then anesthetized and fasting blood samples were collected. Serum was separated and stored at −70 °C till analysis. For retinal studies, only group 1, 3, and 4 were utilized. Retinas were dissected and isolated immediately, rinsed in ice-cold saline, and kept at −70 °C until analysis. All experimental procedures and protocols including anesthesia were in accordance with the Association for Research in Vision and Ophthalmology (ARVO) guidelines to the Care and Use of Experimental Animals as well as the Guidelines of the Experimental Animal Care Center, College of Pharmacy, King Saud University, Riyadh, Saudi Arabia. The experimental animal protocol number 251-EACC-2015; dated 2 November 2015 to use male Wistar albino rats for the current study has been approved by the Experimental Animal Care Center Review Board, College of Pharmacy, King Saud University, Riyadh, Saudi Arabia.

### 2.2. Assay of Glucose and Insulin Levels

Serum glucose levels were measured by using a commercially available kit (RANDOX Laboratories Ltd., Crumlin, Antrim, UK), while insulin serum levels were measured by using ELISA kit (BioSource, Europe S.A., Nivelles, Belgium).

### 2.3. Estimation of Thiobarbituric Acid Reactive Substances (TBARS) Levels

The lipid peroxidation products TBARS levels were measured in the retina using a commercially available assay kit (ZeptoMetrix Co., Buffalo, NY, USA). Retinal homogenate was prepared by applying short burst of ultrasonication in the 10 mM 4-(2-hydroxyethyl)-1-piperazineethanesulfonic acid (HEPES) lysis buffer, pH 7.4, containing 100 mM NaCl, 1% triton X-100, 0.2% sodium dodecyl sulfate (SDS), and a protease inhibitor cocktail. The homogenates were then centrifuged at 11,000× *g* for 20 min at 4 °C using an ultracentrifuge. Following the centrifugation, supernatants were separated and collected for TBAR quantification. 100 μL of the supernatant was mixed with 2.5 mL of reaction buffer provided in the kit. The mixture was then heated at 95 °C for 60 min. After cooling and centrifugation, the absorbance of the supernatant was measured using a spectrophotometer. The protein concentrations in each sample were estimated using Lowry method [30].

### 2.4. Glutathione (GSH) Assay

The total GSH levels were measured in the retina of naringenin of treated and untreated diabetic and non-diabetic rats using the method described by Sedlak and Lindsay [31] with slight modification. Retinal homogenate and supernatant were prepared as described above. Retinal supernatant was deproteinized by adding an equal volume of metaphosphoric acid (2.5%, *w*/*v*). After 5 min, the mixture was centrifuged at 12,000× *g* and supernatant collected. In the supernatant, 5 μL of 4 M triethanolamine per 100 μL was added and assay was performed using 50 μL supernatant from the retina. To this mixture, 100 μL of 0.01 M Ellman’s reagent, (5,50-dithiobis-(2-nitro-benzoic acid)) (DTNB) was added. The absorbance of the clear supernatants was recorded to measure the concentration of GSH using spectrophotometer at 412 nm within 5 min. A standard curve of GSH was prepared from 0 to 10 μM.

### 2.5. BDNF Quantification by Enzyme-Linked Immunosorbent Assay (ELISA)

The level of BDNF was measured in the retina using ELISA kits (Quantikine Human Brain-Derived Neurotrophic Factor, R&D Systems, Minneapolis, MN, USA) according to manufacturer’s instruction. Retinal homogenate was prepared as described above. Fifty microliter, twofold-diluted retinal supernatant (150 μL/retina) containing approximately 75–100 μg of protein was used for quantitative determination of BDNF in 96-well ELISA plates. Each assay was performed in duplicate. The actual concentration of BDNF in each sample was calculated using a standard curve. The ELISA plate readings were done using Autobio Labtech Instruments Co., Ltd. (Zhengzhou, China). The protein concentrations in each sample were estimated using the Lowry method [30].

### 2.6. Western Blot Analysis

Western blot was used for the expression of neurotrophic factors, synaptophysin, proapoptotic and anti-apoptotic proteins expression in the retina of control, diabetic and naringenin treated diabetic rats. We analyzed the expression of pro-BDNF, BDNF, TrkB, Bcl-2, Bax and caspase-3 proteins. First, we made retinal tissues homogenate by ultrasonication in the 10 mM HEPES buffer (pH 7.4), containing 100 mM NaCl, 1 mM Na_3_VO_4_, 10 mM sodium pyrophosphate, 10 mM NaF, 2 mM Ethylenediaminetetraacetic acid (EDTA), 1 mM phenylmethylsulfonyl fluoride (PMSF), 1% Triton X-100, 0.2% SDS, and a protease inhibitor cocktail. Samples were centrifuged at 15,000× *g* for 15 min in cooling centrifuge and supernatants collected and the protein concentrations estimated. Protein samples were boiled in Laemmli sample buffer for 5 min, and equal amount of proteins (50 µg/well) were separated on 10–12% SDS-polyacrylamide gels and transferred onto nitrocellulose membranes. After transferring the proteins, the membranes were blocked for 90 min at room temperature with 5% non-fat milk made in Tris-buffered saline containing 0.1% Tween-20 (TBS-T). The membranes were incubated overnight at 4 °C with anti-proBDNF (1 μg/mL; Santa Cruz Biotechnology, Inc., Dallas, TX, USA), anti-BDNF(1 μg/mL; Santa Cruz Biotechnology, Inc., Dallas, TX, USA), anti-TrkB (1 μg/0.5 mL; Cell Signaling Technology, Danvers, MA, USA), anti-synaptophysin (1 μg/mL; Cell Signaling), anti-caspase-3 (1 μg/mL; Santa Cruz Biotechnology, Inc., Dallas, TX, USA), anti-Bcl-2 (1 μg/mL; Santa Cruz Biotechnology, Inc., Dallas, TX, USA), anti Bax (1 μg/0.5 mL; Santa Cruz Biotechnology, Inc., Dallas, TX, USA), and β-actin (1 μg/mL; Sigma-Aldrich comp, St. Louis, MO, USA), primary antibodies. After overnight incubation with primary antibodies, membranes were washed three times with TBS-T (5 min each) and then incubated with their respective secondary horseradish peroxidase-conjugated antibodies (1:2000; Santa Cruz Biotechnology Inc., Santa Cruz, CA, USA) at room temperature for 90 min. Membranes were then washed four times with TBS-T for 5 min each, and the immunoreactivity of bands was visualized on a LI-COR C-DiGit Blot Scanner from Biosciences, Lincoln, NE, USA, using enhanced chemiluminescence (Western blotting luminol reagents (1:1), Santa Cruz Biotechnology, Inc., Santa Cruz, CA, USA). Protein bands were quantified by densitometry analysis using Image-Lab 2.0.1 software (Bio-Rad Laboratories Inc., Hercules, CA, USA). For internal control, membranes were washed and incubated with a mouse monoclonal β-actin antibody (1:2000; Santa Cruz Biotechnology Inc., Santa Cruz, CA, USA), and all the steps were followed as described above.

### 2.7. Statistical Analysis

All statistical analysis was conducted using, Statistical Package for the Social Sciences Version 12 (SPSS 12.0, Chicago, IL, USA). All data are reported as means ± SEM. Analysis of groups were determined using one-way, analysis of variance (ANOVA) with Mann-Whitney test for analysis of pre- and post-treatment measurements between diabetes, control and naringenin treated groups. Statistical significance was accepted when *p* ≤ 0.05.

## 3. Results

### 3.1. Systemic Effects of Naringenin Treatment on Body Weight, Glucose and Insulin Levels in the Control and Diabetic Rats

Analyses of body weight, glucose and insulin levels in control and diabetic rats were conducted after naringenin treatments. As shown in Table 1, the level of fasting glucose was increased while both insulin levels and body weight were decreased in diabetic rats as compared to control. Oral treatments with naringenin could not correct the body weights. However, naringenin treatment to diabetic rats showed a significant decrease in the levels of fasting blood glucose (250.5 ± 19.5 mg/dL vs. 405.5 ± 21.6 mg/dL; *p* < 0.01) and an increase in the insulin (35.3 ± 3.4 ng/mL vs. 21.3 ± 2.7 ng/mL, *p* < 0.05) compared with untreated diabetic rats. No significant change was observed on body weight, glucose, and insulin levels between the naringenin-treated and non-treated control rats.

### 3.2. Effect of Naringenin on Oxidative Stress in the Rat Retina

**Figure 1:**
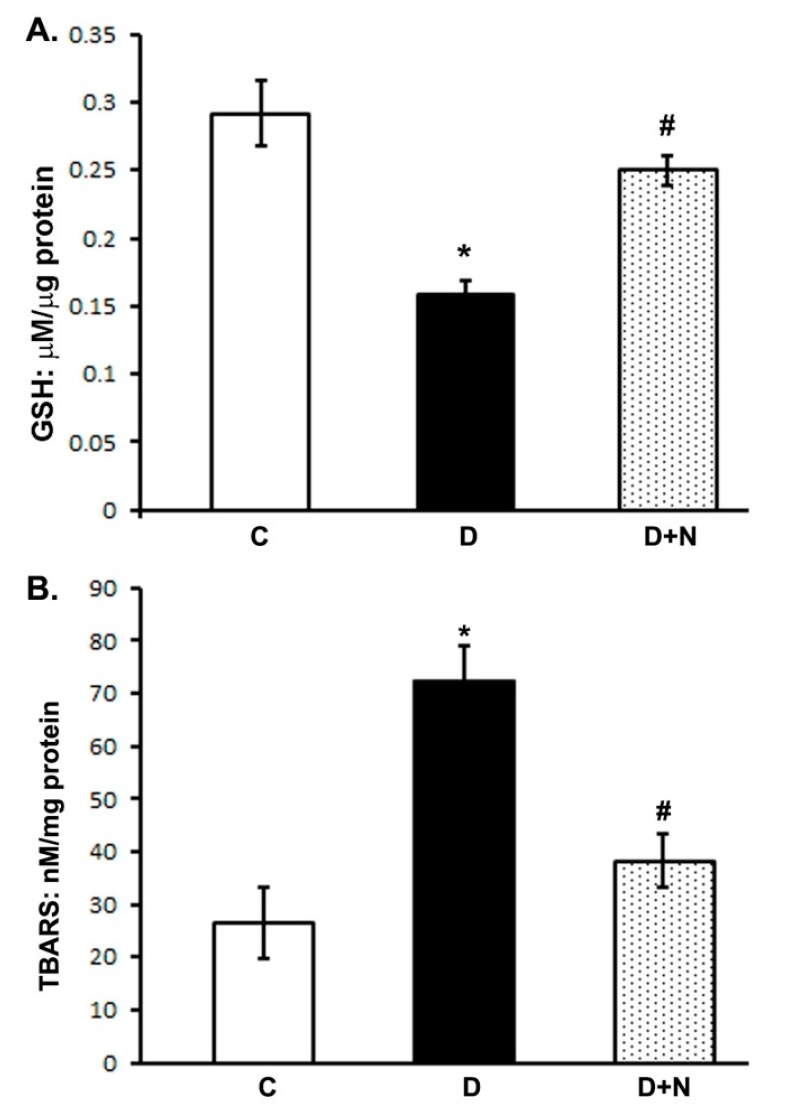
Effects of naringenin on glutathione (GSH) and thiobarbituric acid reactive substances (TBARS) levels in retina of diabetic and non-diabetic animals. (Panel **A**) and (panel **B**) represents measurements of GSH and TBARS respectively. Values are expressed as means ± SEM (standard error of mean); *n* = 6/group. D significantly different from control (C) (* *p* < 0.01); and D + N significantly different from D (^#^ *p* < 0.05). C represents control, D as diabetic, and D + N as diabetic rats treated with naringenin.

We measured the levels of GSH and TBARS, as a marker of oxidative stress in naringenin treated and untreated diabetic rat retinas. The level of GSH significantly decreased (*p* < 0.01) in diabetic retinas as compared to controls. However, naringenin treatments increased the retinal GSH level as compared to non-treated diabetic rats (*p* < 0.05) (Figure 1A). Whereas, the level of TBARS increased at-least three fold in diabetic retinas. Though, naringenin treatments significantly reduced the elevated levels of TBARS in the retina of diabetic rats as compared to non-treated diabetic rats (*p* < 0.05) (Figure 1B).

### 3.3. Effects of Naringenin on Retinal BDNF Levels

**Figure 2:**
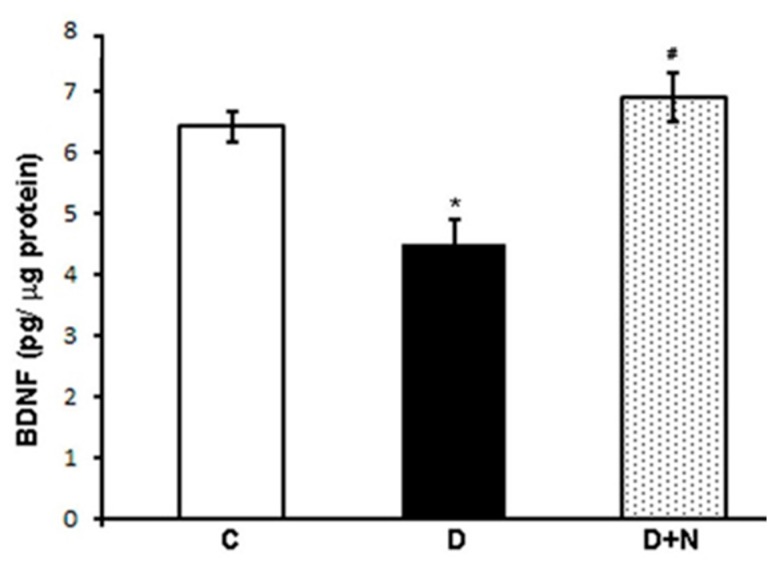
Brain derived neurotrophic factor (BDNF) concentration in the retina of control, naringenin-treated, and non-treated diabetic rats. Values are means ± SEM; *n* = 6/group. *^,#^ *p* < 0.05 compared to control and diabetic rats, respectively. C represents control, D as diabetic, and D + N as naringenin-treated diabetic rats.

The level of BDNF in the retina of diabetic rats was significantly lower as compared to corresponding non-diabetic control group (4.5 ± 0.38 vs. 6.42 ± 0.25 pg/µg protein; *p* < 0.05). However, naringenin treatment significantly increased the level of BDNF in the retina of diabetic rats as compared to untreated diabetic rats (6.9 ± 0.4 vs. 4.5 ± 0.38 pg/µg protein), *p* < 0.05) (Figure 2).

### 3.4. Effects of Naringenin on ProBDNF and BDNF Protein Expression Levels in the Diabetic Rat Retinas

**Figure 3:**
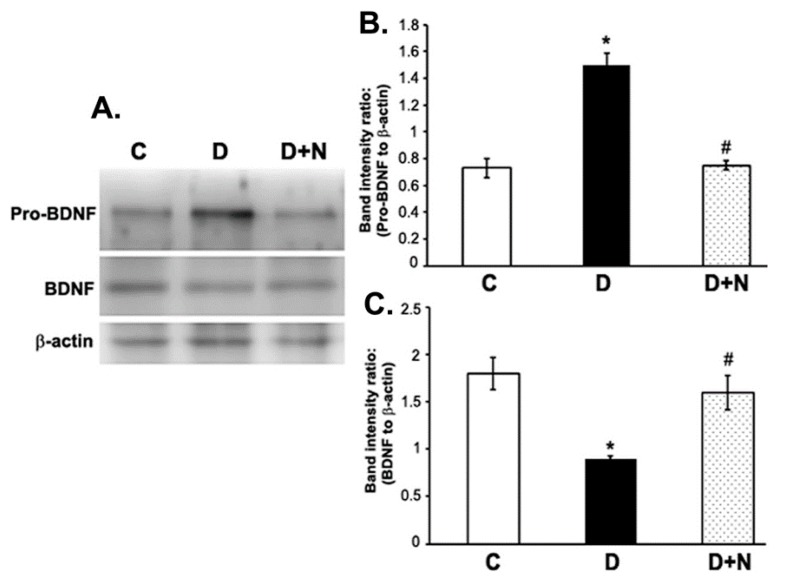
Western blot analysis of the expression of pro-BDNF and BDNF in retinas from control, diabetic, and naringenin-treated diabetic rats. The intensities of the bands were quantified by densitometry. (**A**) Representative immunoblots of pro-BDNF, BDNF and β-actin bands; (**B**,**C**) Data presented as ratios of those protein bands to β-actin. Values are means ± SEM; *n* = 6/group. * *p* < 0.05, significantly different from their controls; ^#^ *p* < 0.05, significantly different from diabetic. BDNF (brain derived neurotrophic factor), pro-BDNF (precursor of brain derived neurotrophic factor). C represents control, D as diabetic, and D + N as diabetic rats treated with naringenin.

Western blotting analysis showed a significant reduction in the expression level of retinal BDNF while pro-BDNF level increased in the retina of diabetic rats compared to non-diabetic control group. However, naringenin treatment to diabetic rats caused a significant reduction in the level of proBDNF and improved the BDNF level to almost control level in the retina of diabetic rats (*p* < 0.05) (Figure 3).

### 3.5. Effects of Naringenin on TrkB and Synaptophysin Protein Expression Levels in the Diabetic Rat Retinas

**Figure 4:**
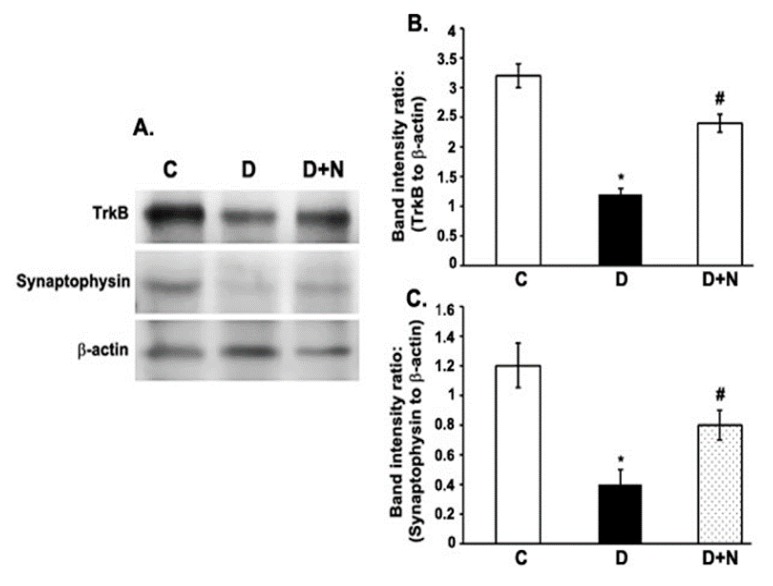
Western blot analysis of the expression of TrkB and synaptophysin in retinas from control, diabetic, and naringenin-treated diabetic rats. The intensities of the bands were quantified by densitometry. (**A**) Representative immunoblots of TrkB and synaptophysin and β-actin bands; Panel (**B**,**C**) Data presented as ratios of those protein bands to β-actin. Values are means ± SEM; *n* = 6/group. * *p* < 0.05, significantly different from their controls; ^#^ *p* < 0.05, significantly different from diabetic. TrkB; Tropomyosin receptor kinase. C represents control, D as diabetic, and D + N as diabetic rats treated with naringenin.

The expression levels of both TrkB (the specific receptor of BDNF) and synaptophysin were significantly decreased in the retina of diabetic rats compared to non-diabetic control group. However, the decreased levels of TrkB and synaptophysin in diabetic retinas were improved by naringenin treatment (Figure 4).

### 3.6. Effects of Naringenin on the Protein Expression Levels of Bcl-2, Bax and Caspase-3 in the Diabetic Rat Retinas

**Figure 5:**
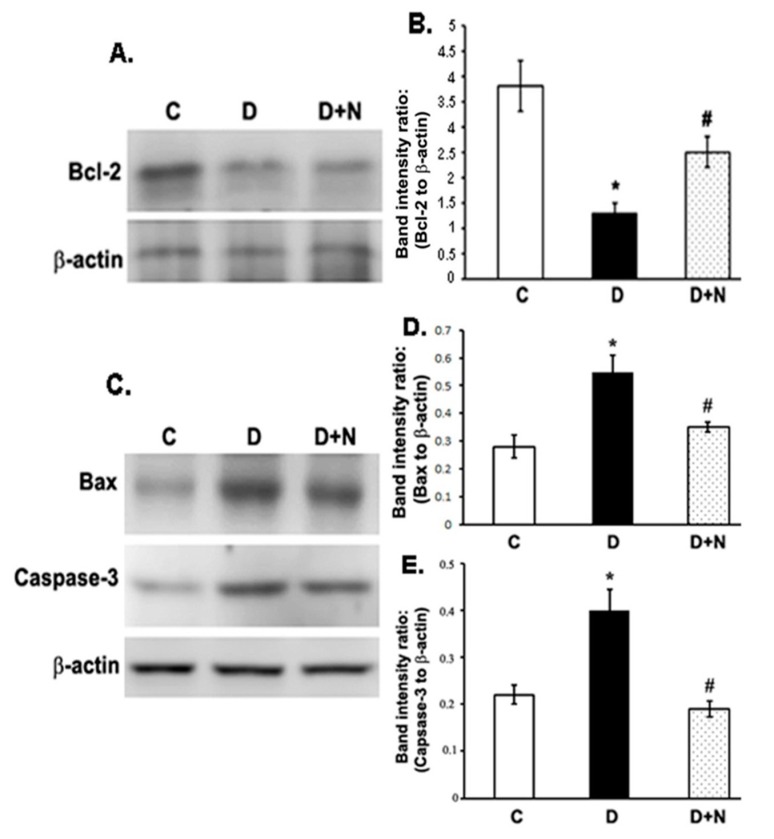
Western blot analysis of the expression of Bcl-2, Bax and caspase-3 in retinas from control, diabetic, and naringenin-treated diabetic rats. (**A**) Representative immunoblots of Bcl-2 and β-actin bands; (**C**) Representative immunoblots of Bax, caspase-3 and β-actin bands; (**B**,**D**,**E**) Data presented as ratios of those protein bands to β-actin. The intensities of the bands were quantified by densitometry. Values are means ± SEM; *n* = 6/group. * *p* < 0.01, significantly different from their controls; ^#^ *p* < 0.05, significantly different from diabetic. Bcl-2; B cell lymphoma 2. Bax; Bcl-2 associated X protein. C represents control, D as diabetic, and D + N as diabetic rats treated with naringenin.

The expression levels of anti-apoptotic Bcl-2 reduced significantly in the diabetic retina compared to controls (*p* < 0.01). However, the decreased level of Bcl-2 in diabetic retinas was significantly augmented when treated with naringenin (*p* < 0.05) (Figure 5A,B). Conversely, the expression levels of pro-apoptotic Bax and caspase-3 proteins increased significantly in the diabetic retinas as compared to controls (*p* < 0.05) (Figure 5C,D and Figure 5C,E). However, naringenin administration to diabetic rats lowered the levels of both caspase-3 and Bax in the diabetic retina to their control levels (*p* < 0.05).

## 4. Discussion

Naringenin is a flavanone which has been reported to have numerous bioactive effects on human health such as being an antioxidant, an anti-inflammatory, anti-diabetic and anti-neurodegenerative. In this study, we analyzed the potential ameliorative effects of the oral treatment of naringenin in the diabetic retina, especially at the levels of oxidants, caspases and neurotrophic factors; whose dysregulated levels have been found to be damaging in the diabetic retina.

Hyperglycemia and decreased levels of insulin have been considered major factors to cause DR [4,32]. As expected, we found increased level of serum blood glucose and a decreased level of insulin in diabetic rats. Interestingly, treatment of diabetic rats with naringenin exhibited remarkable improvement in insulin and lowering of blood glucose levels. Our results are well supported by a number of previous studies that naringenin protected islets against streptozotocin induced oxidative stress by scavenging free radicals and thereby stimulating the remaining pancreatic β cells to synthesize insulin [33,34,35]. Hyperglycemia increases the production of reactive oxygen species and depletes cellular antioxidant defense capacities, that contributes towards the cell death and retinal dysfunction in diabetes [8,36]. In this study, we observed a significant increase in retinal TBAR levels, which is usually a standard marker for lipid peroxidation, whereas the level of endogenous antioxidant glutathione decreased in diabetic retinas compared with control. However, naringenin treatment markedly decreased retinal TBARS to almost control level and returned the levels of GSH towards their control values, suggesting that naringenin may protect the diabetic retina through the inhibition of lipid peroxidation and restoring antioxidant system. Thus, naringenin being an antioxidant might effectively prevent oxidative damage in diabetic retina similar to as observed in various organs affected by diabetes [37,38].

Oxidative stress induced by diabetes is known to activate apoptosis process in the diabetic retina. The increased expression of Bax and caspase-3 early in diabetic retina are correlated with the acceleration of the neuron cell death and reduction of axonal regeneration, which are reliable markers for apoptosis [39,40]. Consistent with the previous studies, we also found increased expression of both Bax and caspase-3 in the diabetic retina. Interestingly, naringenin treatment efficiently decreased their expression, while the drug increased the expression of the survival factor, Bcl-2 protein in the diabetic retina. Previous studies suggest that naringenin activates apoptotic regulatory proteins such as Akt and induce phosphorylation of Erk1/2 which may play an important role in neuroprotection in case of diabetic retina [41,42]. Recently, Kara et al., reported the neuroprotective effect of naringenin by inhibiting the apoptosis of the retinal cells in reperfusion injury and found this drug better than flavonoid, hesperetin for neuroprotection [28]. Therefore, we speculate that antioxidant activity of naringenin in the diabetic retina may be a useful anti-apoptotic intervention for diabetic retina.

It is well documented that BDNF plays an important role in neuronal survival and maintenance [2]. BDNF exerts its action by interacting with TrkB and the downstream signaling cascades after receptor activation that enhances cell proliferation and survival [43,44]. However, proBDNF the precursor of BDNF stimulates neuronal apoptosis and reduces synaptic transmission via activation of p75 receptor [19]. Previously, we and others reported a significant reduction of neurotrophic factors in the diabetic rat retina coincided with the decreased level of retinal TrkB expression [17,45,46]. Consistent with those studies, we found an increased level of proBDNF and a decreased level of BDNF expression, accompanied by a decreased level of TrkB. Remarkably, naringenin treatment to diabetic rats caused a significant decrease in the level of proBDNF while BDNF and TrkB levels were improved. Our results are supported by few previous studies indicating that flavonoids restore the level of neurotrophic factors in the retina of diabetic rats [11,46] and induce the synthesis and secretion of neurotrophic factors in the brain [16,47]. This suggests further that naringenin may improve neurotrophic factors in diabetic retina, which in turn might protect retinal damage.

Synaptophysin is the major synaptic protein necessary for neurotransmission, which is a well-known marker for neurodegeneration in various neurological diseases [48]. Maintaining the level of synaptophysin is necessary for normal synaptic functions such as exocytosis, synaptic vesicle formation, synaptic plasticity, and neurotransmitter delivery [49]. Consistent with previous studies, we found a decreased level of synaptophysin in the diabetic rat retina, which may cause neurodegeneration in the retina [15,50]. Naringenin treatment, significantly increased the level of synaptophysin in the retina of diabetic rats. Our results are supported by Sasaki et al., that antioxidant lutein attenuated oxidative stress and increased the level of synaptophysin in diabetic mice in an attempt to protect retinal neurons [15]. In addition, numerous studies found beneficial effects of flavonoids by protecting the synaptic structure and function in the brain by promoting the expression of synaptophysin [51,52]. Therefore, additionally naringenin might also exert neuroprotective action in the diabetic retina by inducing synaptophysin expression.

## 5. Conclusions

**Figure 6:**
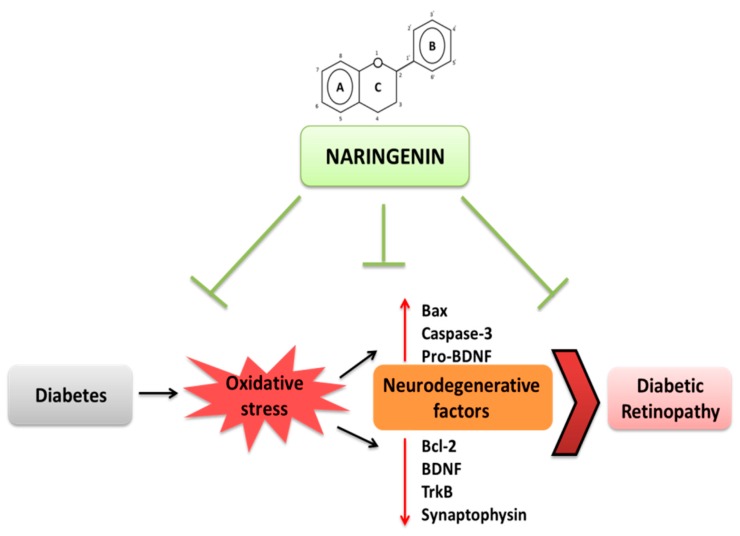
Schematic diagram summarizes diabetes-induced oxidative stress that leads to diabetic retinopathy. The findings of the current study showing that diabetes-induced oxidative stress activates apoptosis and decreases neurotrophic support leading to neuronal damage. Treatments with naringenin that target diabetes, oxidative stress and neurodegenerataive factors might be effective therapeutics in attenuating retinal neurodegeneration in diabetic retinopathy. Bcl-2; B cell lymphoma 2. Bax; Bcl-2 associated X protein. TrkB; Tropomyosin receptor kinase. BDNF; brain derived neurotrophic factor.

The results of our current study demonstrates the beneficial effects of naringenin that possesses anti-diabetic, antioxidant and antiapoptotic properties, which may limit neurodegeneration by providing neurotrophic support to prevent retinal damage in diabetic retinopathy as summarized and depicted in Figure 6. Thus, treatments with naringenin might be effective therapeutics in attenuating retinal neurodegeneration in diabetic retinopathy.

## Figures and Tables

**Table 1:** Effects of naringenin on body weight, blood glucose and serum insulin levels in the rats group as indicated.

	Control	Control + Naringenin	Diabetic	Diabetic + Naringenin
Body weight (gm)	325 ± 16.4	332.2 ± 15.5	225.6 ± 12.3 *	215.7 ± 11.8
Glucose (mg/dL)	92.6 ± 5.6	95.2 ± 6.2	405.5 ± 21.6 *	250.5 ± 19.5 ^#^
Insulin (ng/mL)	45.4 ± 5.4	42.6 ± 3.5	21.3 ± 2.7 *	35.3 ± 3.4 ^#^

Body weights, glucose and insulin were measured five weeks after naringenin treatments. The values are means ± SEM (standard error of mean) of 7–10 rats in each group. (* *p* < 0.01; diabetic vs. control); (^#^ *p* < 0.05; diabetic + naringenin vs. diabetic).
